# Fabrication of an rhBMP-2 loaded porous β-TCP microsphere-hyaluronic acid-based powder gel composite and evaluation of implant osseointegration

**DOI:** 10.1007/s10856-014-5250-0

**Published:** 2014-06-14

**Authors:** Jae Hyup Lee, Jungju Kim, Hae-Ri Baek, Kyung Mee Lee, Jun-Hyuk Seo, Hyun-Kyung Lee, A-Young Lee, Guang Bin Zheng, Bong-Soon Chang, Choon-Ki Lee

**Affiliations:** 1 Department of Orthopedic Surgery, College of Medicine, SMG-SNU Boramae Medical Center, Seoul National University, 425 Shindaebang-2-Dong, Seoul, 156-707 Korea; 2 Institute of Medical and Biological Engineering, Medical Research Center, Seoul National University, Seoul, 110-799 Korea; 3 Research Center, Bioalpha, Seong-Nam, 462-120 Korea; 4 Bio Division, Daewoong Pharmaceuticals, Seoul, Korea; 5 Department of Orthopedic Surgery, College of Medicine, Seoul National University Hospital, Seoul, 110-744 Korea

## Abstract

Methods to improve osseointegration that include implantation of rhBMP-2 with various kinds of carriers are currently of considerable interest. The present study was conducted to evaluate if the rhBMP-2 loaded β-TCP microsphere-hyaluronic acid-based powder-like hydrogel composite (powder gel) can act as an effective rhBMP-2 carrier for implantation in host bone with a bone defect or poor bone quality. The release pattern for rhBMP-2 was then evaluated against an rhBMP-2-loaded collagen sponge as a control group. Dental implants were also inserted into the tibias of three groups of rabbits: an rhBMP-2 (200 µg) loaded powder gel composite implanted group, an implant only group, and a powder gel implanted group. Micro-CT and histology of the implanted areas were carried out four weeks later. The rhBMP-2 powder gel released less rhBMP-2 than the collagen sponge, but it continued a slow release for more than 7 days. The rhBMP-2 powder gel composite improved osseointegration of the dental implant by increasing the amount of new bone formation in the implant pitch and it improved the bone quality and bone quantity of new bone. The histology results indicated that the rhBMP-2 powder gel composite improved the osseointegration in the cortical bone as well as the marrow space along the fixture. The bone-to-implant contact ratio of the rhBMP-2 (200 µg) loaded powder gel composite implanted group was significantly higher than those of the implant only group and the powder gel implanted group. The powder gel appeared to be a good carrier and could release rhBMP-2 slowly to promote the formation of new bone following implantation in a bone defect, thereby improving implant osseointegration.

## Introduction

The screw type metal implant is widely used in orthopedic, dental, plastic, and maxillofacial surgery. As the number of elderly people has increased, the rate of dental implantation has risen and surgery for patients that have poor bone stock has also been increased. Osseointegration of a metal implant is greatly influenced by the quality of the host bone, and it can be negatively affected by previous radiation therapy or infection, smoking, bone defects, osteoporosis, etc., thereby endangering the survival of the implant [1–4]. For patients with bone defect or poor bone stock, successful dental implant surgery could increase the bone quantity and quality of the implantation site, thereby improving the osseointegration. Therefore, methods to strengthen osseointegration that include implantation of a growth factor to promote bone healing are currently of considerable interest.

Recombinant human bone morphogenetic protein-2 (rhBMP-2), a member of the transforming growth factor beta superfamily, has been widely studied as a treatment for bone healing due to its excellent osteoinductivity. Growth factors like rhBMP-2 can be delivered to bone defects by several delivery vehicles, including ceramics, polymers, and composites. For dental implant fixation on the implantation site in patients with bone defect or poor bone quality an injectable rhBMP-2 carrier is needed.

Inorganic carriers, such as a tricalcium phosphate (TCP) microsphere have the advantage of being structurally similar to bone, but they also have the disadvantage of being difficult to mold to the shape of a defect; they also have difficulty maintaining strong cohesion [5]. In contrast, many natural polymers have the advantages of being biocompatible, biodegradable, and soluble in physiologic fluids; however, natural polymers derived from animals have risks in terms of immunogenicity or pathogen transmission [6]. These limitations have been overcome by using composites that combine the advantages of the ceramic vehicles, such as osteoconductivity, and the advantages of the natural polymer families, which are moldable and have biodegradable characteristics [7].

Hyaluronic acid (HA) is one of the major components of the extracellular matrix (ECM) and it is found in all the connective tissues of the body. It is a naturally-derived, linear, high molecular weight polymer with viscoelastic properties [8]. HA is an important element that functions in major biological processes, such as tissue organization, wound healing, angiogenesis, and remodeling in skeletal biology [9–12]. In addition, HA is anionic and therefore capable of forming ionic bonds with cationic growth factors like rhBMPs, which is of significance for clinical applications [13]. In addition, HA is injectable, plays a role as an rhBMP-2 carrier, and has the advantage of requiring no premixing during the surgical operation [14]. Although HA hydrogel has the disadvantage of being too weak to hold mechanical loading, it helps increase loading-bearing prosperity through chemical modification and cross-linking. Other natural polymers with potential use in rhBMP delivery, such as gelatin, dextran, and fibrin, are limited by their insufficient mechanical strengths [15].

Inorganic materials used as rhBMP-2 delivery vehicles include β-TCP, which has rhBMP-2-binding activity and which is a useful carrier of rhBMP-2 because of its osteoconductivity and its rapid resorption in the body [5]. One study reported that it recovered a bone defect faster than an autologous bone graft due to carrier characteristics that aided the sustained release of rhBMP-2 in the bone defect [16].

The cross-linked hyaluronic acid powder gel used in the present study has the property of absorbing fluids, like blood generated from damaged bone tissue, and it has the advantage that its swelling property (its ability to absorb fluids) and its biocompatibility are not readily changed by the surrounding environment due to its cross-linking. A complex carrier consisting of a mixture of cross-linked powder gel and β-TCP microspheres shows good absorption and it can deliver rhBMP-2 during osteogenesis because its physical characteristics are not appreciably changed; consequently, it promotes bone regeneration around an implant.

For effective delivery of rhBMP-2, inorganic β-TCP microspheres were combined with an HA-based powder-like hydrogel, a natural polymer referred to as a “powder-gel” in this study. The aim of the present study was to evaluate if the powder gel composite showed slow release of rhBMP-2 and if it could act as an effective rhBMP-2 carrier for implantation in host bone with a bone defect or poor bone quality. In addition, the rabbit tibia implantation model was used to determine if the powder gel composite loaded with rhBMP-2 could strengthen the osseointegration of a dental implant.

## Materials and methods

### Injectable rhBMP-2 carrier fabrication and an rhBMP-2 release test

#### Porous β-tricalcium phosphate microsphere

**Fig. 1:**
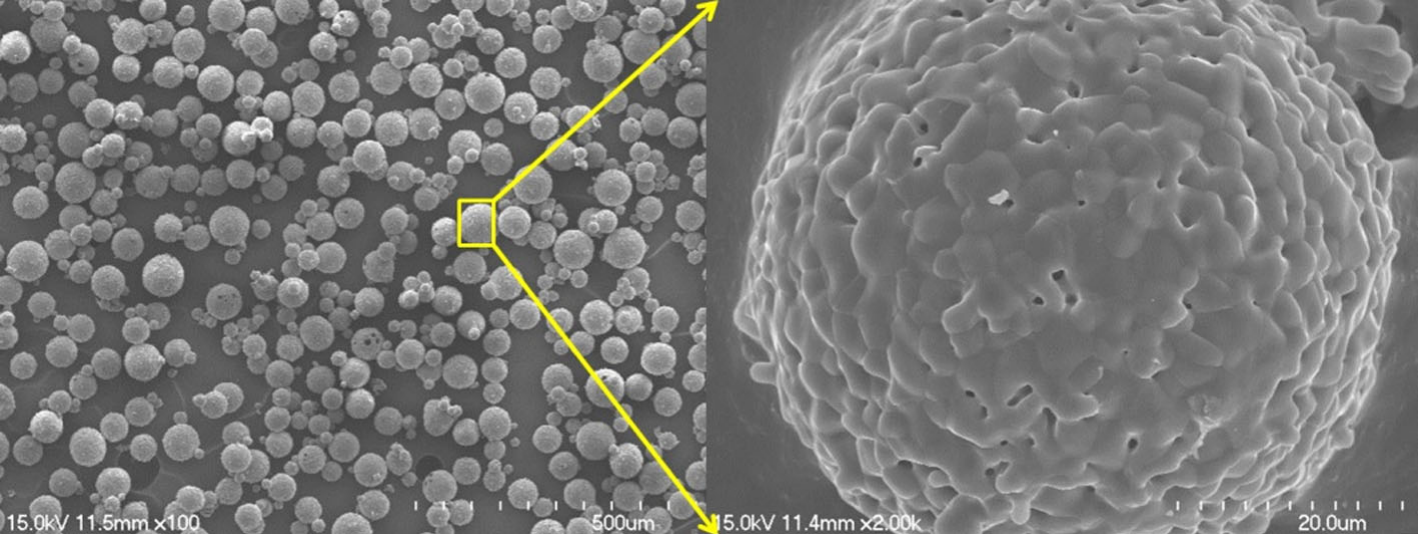
SEM images of β-TCP microspheres generated by the spray-dry method after sintering at 1,250 °C

The porous β-TCP (Cerectron Co., Kimpo, South Korea) microspheres were prepared by the spray-dry method. The resulting spherical particles were subsequently sintered at 1,250 °C for 2 h, resulting in diameters ranging from 45 to 75 μm (Fig. 1). The β-TCP that was sintered at high temperature had a porosity of 59.3 %, as measured with a mercury porosimeter.

#### Hyaluronic acid (HA)-based powder gel

The powder gel was prepared to facilitate the loading of rhBMP-2 and to improve the fixation force of the carrier at the site of a bone defect by absorbing the blood and body fluids generated at the defect site. The powder gel was a cross-linked hyaluronic acid-based hydrogel consisting of HA (Bioland Co., Ochang, South Korea) with a high molecular weight of 3 million Daltons. This HA-based hydrogel was prepared through cross-linking by adding butanediol diglycidyl ether (Sigma-Aldrich, St. Louis, USA), a cross-linking agent, to 2.7 wt% of HA solution [17]. The remaining reagents were removed without resuspending by dialysis against 1X PBS (Sigma-Aldrich, USA) for 5 days. The resulting HA-based hydrogel was lyophilized for 4 days and then ground and sieved to yield a gel powder with particles less than 100 µm in diameter.

#### Carrier preparation

Conformational change of the rhBMP-2 due to sterilization was avoided by mixing the rhBMP-2 solution with a powder gel composite to a final concentration of 1 mg/ml rhBMP-2. The in situ mixing process is a method used to generate an rhBMP-2 loaded injectable carrier by mixing the powder gel and the porous spherical particles for loading rhBMP-2.

In other words, one syringe contained porous β-TCP microspheres and the other syringe held the HA-based powder-gel containing 1 mg/ml rhBMP-2, and the relative weight ratio of the materials was 1:9. These two syringes were interconnected through a 2-way connector and 30 cycles of piston movement of each syringe were performed to ensure that the mixture was homogeneous. Collectively, β-TCP microspheres and HA were used as an rhBMP-2 carrier in this study.

#### Analysis of the physical properties of the HA-based powder gel

The reticular microstructure of the HA-based powder gel was induced by cross-linking and analyzed using a scanning electron microscope (SEM, S-4700, Hitachi, Tokyo, Japan). The swelling properties of the HA-based powder gel were measured by incubating it in PBS at room temperature. The swelling ratio was measured by comparing the change in the wet weight of the hydrogel before and after 3 days of incubation. The percentage of water absorbed (Wa) was calculated by the following formula: Swelling ratio (%) = ((Ww-Wi)/Wi) X 100 % (Ww: wet weight of hydrogel; Wi: initial weight of hydrogel).

#### rhBMP-2 release rate study

The release rate for rhBMP-2 was evaluated by impregnating rhBMP-2 into the hyaluronic acid-based powder gel composite. The experiment was carried out by impregnating rhBMP-2 into the composite and then analyzing the rhBMP-2 release, as detected by an antigen-antibody reaction and ELISA analysis. A collagen type I sponge (Bioland, Ochang, South Korea), which is currently used as a periodontal tissue regeneration-inducing agent in dentistry, was impregnated with 100 µg rhBMP-2 and used for comparison. The release patterns from the sponge and the composite were analyzed following treatment with hyaluronidase (100 units/ml) and collagenase (20 CDU/ml) (Sigma, USA) for 7 days, and incubation at 37 °C in PBS [18, 19].

### In vivo study

#### Animals and implantation

**Fig. 2:**
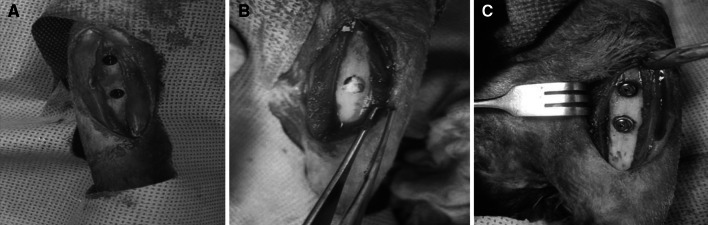
Surgical procedures. **a** Tibial diaphysis in the tibial medial aspect was exposed and two, 4 mm diameter holes were generated; **b** A composite of rhBMP-2 loaded porous β-TCP microspheres and hyaluronic acid powder-gel was injected;** c** A dental implant fixture was inserted

A total of 17 New Zealand white male rabbits (3–3.5 kg) underwent general anesthesia with Zoletil^®^ and xylazine. This study was approved by the Institutional Animal Care and Use Committee at the Clinical Research Institute of Seoul National University Hospital Biomedical Research Institute (IACUC No. 13-0038). The hind limb was disinfected with betadine after hair removal. The anterior aspect of the tibia was exposed and two holes were generated by pre-drilling. The two holes were spaced no less than 15 mm apart in order to minimize the interaction between them. After the two holes for the implant fixture in the tibia were placed using a lance drill, they were enlarged to a diameter of 2 mm using a twist drill. Next, a straight drill was used to further enlarge each of the holes first to 2.8 mm, then to 3.3 mm, and finally to 3.8 mm; and then the holes were countersinked. The depth of each of the holes was 8.5 mm and dental implant fixtures, 4 mm in diameter, (Ø4, 8.5 mm length, MegaGen, Seoul, South Korea) were inserted. The insertion torque of the implant was 45 Ncm. Three groups were allocated as follows: the implant only group (implant group), the injected β-TCP-hydrogel power gel composite implant group (the hydrogel group), and the rhBMP-2 (200 μg) loaded powder gel β-TCP-hydrogel composite implant group (BMP-2 group). After two tibiae of a rabbit were exposed, two implants of the same group were inserted per each tibia. The block randomization was used to decide which implant to place into each tibia. After inserting a cover screw, and fascia layer, the wound was then sutured (Fig. 2). Immediately after the surgery, 300 mg cefazolin was injected intramuscularly and antibiotics were administered for 2 days. The experimental animals were euthanized 4 weeks later.

### Evaluation methods

#### Plane radiographs

The experimental animals were sacrificed and plane radiographs of the full length of the tibia were obtained at 45 kV for 12 ms. The implants were evaluated for loosening, pullout, or differences in the surrounding osseous tissue.

#### Micro-CT evaluation

The experimental animals were sacrificed and micro-CT was performed on all specimens by harvesting samples that included the implanted part of the tibia. Micro-CT (SkyScan 1173, Zwijnaarde, Belgium) was performed using an aluminum filter in a mid-resolution of 20 μm at 30 kV, 60 μA. The newly formed bone area ratio (the ratio of the area of new bone formation at the space between two threads in the pitch) and the bone-to-implant contact ratio (the ratio of bone directly attached to the thread of the screw) were evaluated. The quality of new bone was evaluated based on a total of 42 pictures analyzed by CTAn, a micro-CT analysis program. The bony tissue of the new bone was analyzed with an ROI (region of interest) at a width of 0.99 mm and a height of 2.48 mm. The bone fraction directly attached to the screw thread was analyzed with an ROI at a width of 0.97 mm and a height of 2.48 mm. For quantification of the quantity and quality of the new bone, the following items were evaluated: percent bone volume (bone volume/trabecular volume), specific surface (bone surface/volume ratio), trabecular bone pattern factor (the parameter of the connectedness of these bone patterns), trabecular thickness (the thickness of the trabeculae), trabecular number (the number of trabeculae), and trabecular separation (the distance between the trabeculae).

#### Histologic evaluation

The full length of the tibia specimen was fixed in formalin for 5 days and the tibiae containing each specimen were divided into two parts. Gross section tissue was put into a cassette and washed for 6 h and then dehydrated in 100 % alcohol. The tissue was put into methacrylate-based chemical curing resin and stirred for 2 days, and then it was stirred and embedded by dissolving in benzoyl peroxide. The block was trimmed and sectioned along the longitudinal axis of the dental implant fixture using an EXAKT cutting instrument (BS-3000 N). Moreover, a 4 μm section was made at the right center of the implant along the sagittal plane and included the surrounding tibia. Grinding was carried out using an EXAKT grinding machine (4,110), and an acrylic slide attachment was performed. The slide was stained with hematoxylin and eosin (H&E) staining and viewed with a light microscope to determine the bond between the bone and the implant and the new bone formation around the implant. Histomorphometric evaluations of the dental implant fixtures were carried out after a scaled calibration using a morphometry program (LEICA IM50 Image Manager, version 4.0). The bone-to-implant contact ratio was measured in the marrow space of the tibia. All measurements were performed using a (X12.5) magnification objective.

### Statistical analysis

The three groups were compared using a nonparametric Kruskal–Wallis test. *P* values < 0.05 were considered significant.

## Results

### Physical properties of the HA-based powder gel

**Fig. 3:**
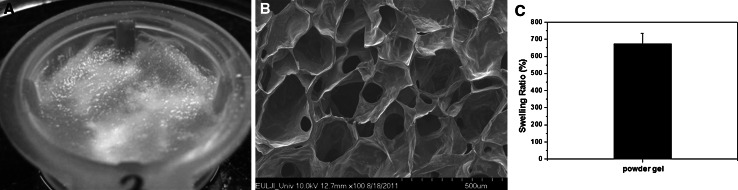
**a** SEM images of cross-linked and freeze-dried HA-based hydrogel; **b** Images of the hydrated HA-based powder-gel; **c** Swelling ratio of the HA-based powder-gel

The prepared HA-based powder gel was uniformly rehydrated (Fig. 3a). A SEM image of cross-linked and freeze-dried HA-based hydrogel is presented in Fig 3b. The HA-based powder gel showed a swelling ratio of 671.4 % in PBS (Fig. 3c), which means that, in this study, the powder gel has sufficient capacity to load the BMP-2 solution. The HA-based powder gel was mixed with β-TCP spherical powder using the in situ mixing process described above.

#### rhBMP-2 release results

**Fig. 4:**
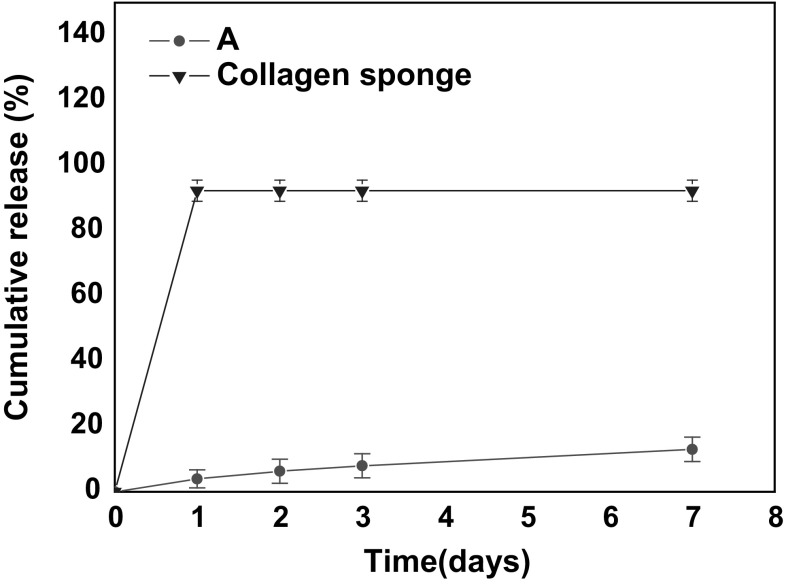
In vitro release of BMP-2 (*A*: Hyaluronic acid (HA)-based powder-gel; Control: collagen sponge)

The collagen sponge was completely decomposed in 1 day and the impregnated rhBMP-2 was fully released within 1 day (Fig. 4). In contrast, the hyaluronic acid-based powder gel composite showed slow release compared to the collagen sponge, and the amount of rhBMP-2 released was less than 20 % of the total after 7 days.

### In vivo results

#### Gross specimens and plane radiographic results

**Fig. 5:**
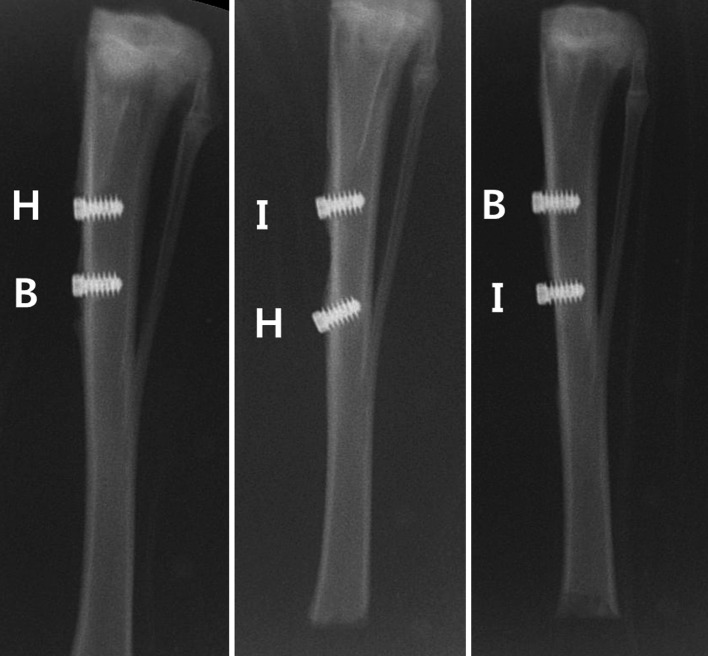
Plane radiographs. *H*: A composite of the β-TCP microspheres and the hyaluronic acid powder gel was injected and a dental implant fixture was inserted (hydrogel group); *B*: A composite of the rhBMP-2 loaded porous β-TCP microspheres and the hyaluronic acid powder gel was injected and then a dental implant fixture was inserted (BMP-2 group). *I*: Only a dental implant fixture was inserted (implant group)

No cases of death occurred in the experimental animals and no infection or inflammation was observed in 17 rabbits. The implant group showed no osseous tissue around the implants, but the BMP-2 group showed newly formed bone wrapped around the head of the implanted fixture. No loosening or pullout of the fixture was observed in the X-rays of either group (Fig. 5).

#### Micro-CT results

**Fig. 6:**
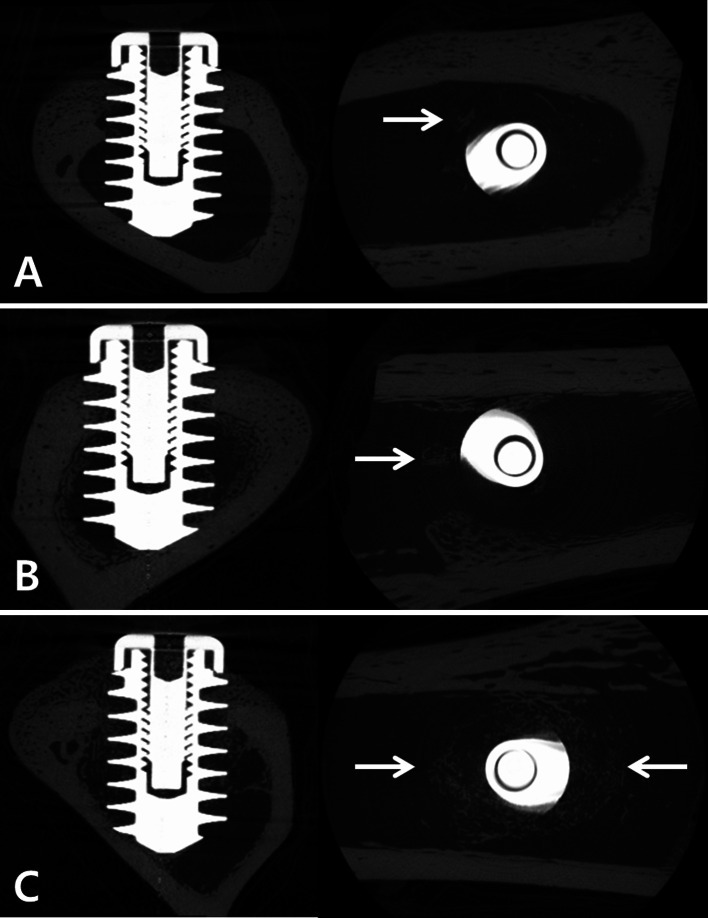
Micro-CT results. **a** Only a dental implant fixture was implanted (implant group); **b** A composite of the β-TCP microspheres and the hyaluronic acid powder gel was injected and a dental implant fixture was inserted (hydrogel group); **c** A composite of the rhBMP-2 loaded β-TCP microspheres and the hyaluronic acid powder gel was injected and a dental implant fixture was inserted (BMP-2 group). In panel **a**, new bone formation was insignificant around the implant fixture but some new bone was shown in panel **b**, while extensive new bone formation was observed in panel **c** (*arrows*: newly formed bony tissue)

The micro-CT results for the implant group revealed a scanty amount of newly formed bone around the fixture in the marrow space. The hydrogel group showed some newly formed bone around the fixture, but the amount was negligible. On the other hand, the BMP-2 group showed excellent osseointegration between the cortical bone and the implant and newly formed bone was identified in the marrow space around the fixture (Fig. 6). The bone-to-implant contact ratio within the fixture pitch of the implant group and the hydrogel group were 6.93 ± 3.8 % (n = 20) and 4.90 ± 7.8 % (n = 20), respectively. By comparison, the ratio for the BMP-2 group was 18.5 ± 10.1 % (n = 20) and this value was significantly higher than that of the other two groups (*P* = 0.0043 and 0.0001 for the implant group and the hydrogel group, respectively).

The newly formed bone area ratio generated within one pitch of fixture of the BMP-2 group (13.9 ± 9.9 %) was significantly higher than that of the implant group (2.1 ± 4.2 %) or the hydrogel group (1.0 ± 1.5 %) (*P* < 0.0001 in both cases).

For the newly formed bone, the percent bone volume, trabecular thickness, and trabecular number were significantly higher in the BMP-2 group than in the implant group (Table 1). Trabecular separation and the trabecular pattern factor were significantly lower, indicating that the quantity and the quality of the formed bone were improved in the BMP-2 group in comparison to the implant group.

**Table 1:** Micro-CT results at 4 weeks after the implantation. The BMP-2 group showed significantly higher percent bone volume, trabecular number, trabecular thickness than the Implant group

Group (n)	BV/TV	BS/BV	Tb.Pf	Tb.Th	Tb.N	Tb.Sp
	Average (std)					
Implant group (30)	5.6 (1.4)	171.6 (21.0)	19.4 (7.4)	0.034 (0.007)	1.64 (0.27)	0.20 (0.04)
Hydrogel group (30)	6.7 (3.2)	167.2 (24.2)	19.4 (9.5)	0.035 (0.006)	1.89 (0.60)	0.18 (0.05)
BMP-2 group (30)	10.4 (5.3)	146.0 (32.6)	9.4 (13.5)	0.042 (0.009)	2.39 (0.85)	0.16 (0.04)
*P* value	<0.0001	0.0006	0.0007	0.0002	<0.0001	0.002

*P*-value: Implant group and BMP-2 group

*BV*/*TV* Bone Volume/Trabecular Volume, percent bone volume

*BS*/*BV* Bone Surface/Bone Volume ratio, specific surface

*Tb.Pf* Trabecular Pattern factor

*Tb.Th* Trabecular Thickness

*Tb.N* Trabecular Number

*Tb.Sp* Trabecular Separation

#### Histologic results

The bone-to-implant contact ratio within the marrow space of the tibia of the implant group, the hydrogel group, and the BMP-2 group was 8.3 ± 2.1 % (n = 6), 7.6 ± 3.8 % (n = 6), and 23.2 ± 6.4 % (n = 6) respectively. The bone-to-implant contact ratio for the BMP-2 group was significantly higher than the ratios of the implant group and the hydrogel group (*P* = 0.0003 and 0.0004, respectively).

**Fig. 7:**
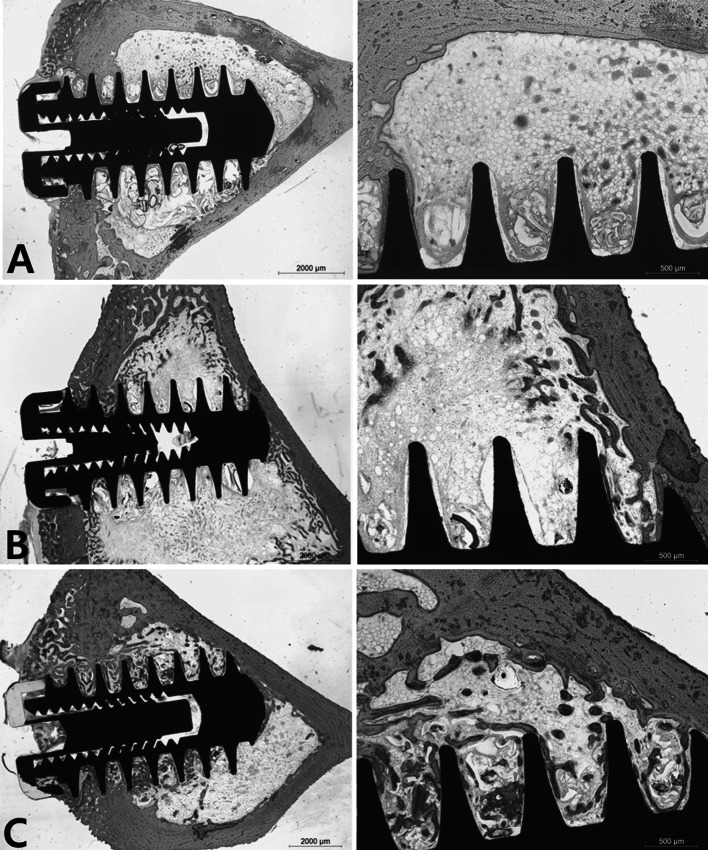
Undecalcified histology. The image magnification on the left and right was ×12.5 and ×40, respectively. **a** Only a dental implant fixture was implanted (implant group). Bone coupling with cortical bone was stable, but the osseous tissue associated with the fixture screws in the marrow space was insignificant; **b** A composite of the β-TCP microspheres and the hyaluronic acid powder gel was injected and a dental implant fixture was inserted (hydrogel group). Bone bonding in the cortical bone was also robust, but new bone formation in the marrow space was insignificant even in comparison to the implant group. The tissue within the pitch is composed of fibrous tissue and exists as intervening tissue between the bony tissues of the marrow space; **c** A composite of the rhBMP-2 loaded β-TCP microspheres and the hyaluronic acid powder gel was injected and a dental implant fixture was inserted (BMP-2 group). The bone bonding in the cortical bone was stable and osseous tissue was connected from the cortical bone to the marrow space. Newly formed bone was evident around the fixture within the marrow space, and newly formed bone was fused with the fixture

The undecalcified histology results indicated that the implant group showed stable fixation between the fixture and the cortical bone, but only a small amount of new bone formation around the fixture was observed in the tibia marrow space (Fig. 7). The hydrogel group also showed good osseointegration between the fixture and the cortical bone, but even less new bone formation was evident around the fixture in the tibia marrow space than was seen in the implant group. The intervening fibrous tissue found within the pitch and within the marrow space suggested that the injected composite became fibrous tissue without forming bone. In comparison, the BMP-2 group showed strong ossetointegration in the cortical bone and osseous tissue was connected to the marrow space along the fixture. Newly formed bone was evident around the fixture in the marrow space and remnant hydrogel powder was observed where new bone was formed.

## Discussion

In general, during the dental implant fixation, there should be enough quantity and good quality of an alveolar bone to cover the entire implant for long-term success [20, 21]. However, the number of patients with an alveolar bone that are not suitable for dental implant fixation have increased due to the growing number of elderly people in the population and the need for re-surgery. Thus, the present study was conducted to improve bone formation at a site that has bone defect or insufficient bone quality, and to evaluate dental implant osseointegration using hyaluronic acid, which is soluble and injectable, and a mixture of rhBMP-2 and β-TCP microsphere, which has well-established osteoconductivity and binds well with rhBMP-2.

The hyaluronic acid that was used for this study underwent hydroexpansivity by binding with water molecules [22], and it could be expand thoroughly on the defect area by contact with blood or tissue fluid when it was injected into the bone defect. Moreover, unlike slow degrading hydroxyapatite, the ability of the fast degrading TCP results in the release of calcium ions. Therefore, TCP is known for its osteoinductive ability to trigger the signaling pathway through protein kinase C pathways and promote the gene expression of BMPs [23–25].

One role of rhBMP-2 is to promote differentiation of monocytes or mesenchymal stem cells into chondrocytes by promoting their recruitment or proliferation, and then inducing new bone formation through osteoblastic differentiation [26]. Therefore, the number of mesenchymal stem cells in the host is important. From this aspect, the rabbit tibia diaphyseal implantation model used in this study has the advantage of being able to create a similar environment without causing a bone defect because there is no directly contacted osseous tissue around an implant in the marrow space except for the tibial cortical bone. Since the proximal metaphyseal area of the tibia has thin cortical bone and large portion of cancellous bone, a dental implant can be fixed by cancellous bone around near cortex and marrow space although it is not fixed to the far cortex. So, in the present study, the dental implant was not placed in the metaphysis; rather, it was fixed to the near cortex of the diaphysis without contacting the far cortex after countersinking. Thus, it is more difficult to generate newly formed bone when inserting an implant into the tibial diaphysis than when making a bone defect in the mandible or maxilla and then inserting a dental implant. For this reason, this model has the advantage of being able to reduce experimental errors because it is favorable for evaluating the experimental results and the stability of the implant can be well-maintained by the cortical bone.

One limit of this model is that it is unable to provide an environment that is similar to a bone defect site insertion environment. The powder gel-BMP-2 that was implanted into the tibial diaphyseal intramedullary space could not be contained within the defect area and intramedullary blood was infiltrated into BMP-2, which may affect the bone formation [14]. Even though the porous β-TCP microsphere-hyaluronic acid powder gel composite shows osteoconductivity of β-TCP, the rate of osseointegration and new bone formation in this group was similar to the control group. This is possibly because new bone formation did not easily occur in the animal model used in this study. Therefore, in the present study, the new bone formation using the powder gel-BMP-2 composite was significantly high, indicating that the powder gel performed effectively as a carrier of rhBMP-2.

A fairly long time is required for in vivo bone formation, which means that rhBMP-2 needs to be released continuously [27]. The results of the present study showed that a collagen carrier gave an initial burst of released rhBMP-2 in a period of 1 day, whereas the hyaluronic acid-based powder gel released rhBMP-2 continuously for up to 7 days. A previous report on bone healing revealed that the sustained release of growth factor is more favorable than a burst release [28].

The micro-CT and histomorphometric results showed that the newly formed bone fraction was significantly higher in the BMP-2 group, when compared to the levels in the implant group and the hydrogel group.

These results indicate that the composite was efficacious as a carrier of rhBMP-2 and bone was formed in an adjacent area where rhBMP-2 was directly and in where no host bone exists. The bone quantity and quality in the BMP-2 group were significantly improved when compared to the results from the implant group, as the BMP-2 group showed significantly higher parameters of micro-CT for bone quantity, such as percent bone volume, trabecular thickness, and trabecular number in the newly formed bone, and significantly lower parameters for bone quality, such as trabecular separation and trabecular pattern factor. These results imply that the rhBMP-2 loaded powder gel composite strengthened osseointegration through new bone formation around the dental implant in the rabbit tibia model. In comparison, the hydrogel group showed even lower new bone formation than the implant group (although the difference was not statistically significant), indicating that the porous β-TCP microspheres acted as a barrier between the implant and the tissue surrounding the implant because the hyaluronic acid powder gel only shows osteoconductivity [29–31]. This phenomenon is evident in the histology results. The fact that fibrous tissue formation was observed between the implant and the bone means that, when only β-TCP microspheres and hyaluronic acid powder gel are implanted, the combination may negatively affect the osseointegration of an implant. In contrast, if β-TCP microspheres and hyaluronic acid powder gel are mixed with rhBMP-2 and injected, they are expected to strengthen the osseointegration of the dental implant by promoting significant formation of new bone through their action as carriers of rhBMP-2, even in an environment where a bone defect exists or bone quality is poor.

The rhBMP-2 release experiment showed that the degree of osteoinductivity is difficult to ascertain because less than 20 % of the total rhBMP-2 in the composite was released after seven days. While both of the components in the composite used in this study could act as carriers of rhBMP-2, the β-TCP microspheres are considered to have combined more strongly with rhBMP-2. Because the rhBMP-2 release was relatively small in this study, when compared to rhBMP-2 release kinetics using a hyaluronic acid-based hydrogel which were previously reported [32, 33]. Moreover, the authors have observed that the rhBMP-2 release pattern of the β-TCP microsphere in the present data seemed similar to the pattern in our previous unpublished data. Thus, this indicates that β-TCP binds more strongly with rhBMP-2 and β-TCP is the rate limiting step of the rhBMP-2 kinetic release.

Gelatin release kinetics has indicated that the initial burst of rhBMP-2 is achieved by diffusion [34, 35]. However, in a situation where sustained release primarily occurs, enzymatic degradation is the more important mechanism [35]. As shown in the present study, the porous β-TCP microsphere and hyaluronic acid powder gel composite may not release sufficient amounts of rhBMP-2 in situations where sustained release occurs due to the strong binding of rhBM-2. In fact, the ratio and particle size of the β-TCP microsphere are important in determining the degradation rate [36]. Moreover, the β-TCP used in this study had a particle size ranging from 45 to 75 μm and it was less degraded than a nano-sized β-TCP. Therefore, in order to achieve the maximum efficacy of rhBMP-2, the composite properties must be improved.

However, the powder has been determined to be a long-term delivery carrier while collagen is used as a short-term delivery carrier of rhBMP-2, and the powder would be expected to improve the bone healing rate at the same dose [37].

## Conclusion

This study showed that rhBMP-2 can be released slowly from a composite of β-TCP microspheres and a hyaluronic acid-based powder-like hydrogel. When the composite is used as a carrier for rhBMP-2 and injected together with a dental implant in a rabbit tibia, it significantly increased the formation of new bone within the implant pitch in the marrow space and strengthened osseointegration of the implant.
